# Pharmacokinetics, Tissue Distribution and Excretion of Paeonol and Its Major Metabolites in Rats Provide a Further Insight Into Paeonol Effectiveness

**DOI:** 10.3389/fphar.2020.00190

**Published:** 2020-02-28

**Authors:** Xintong Hu, Liqin Ding, Shijie Cao, Lina Cheng, Kun Wang, Chenxi Guang, Wei Li, Kazuo Koike, Feng Qiu

**Affiliations:** ^1^ School of Chinese Materia Medica, Tianjin University of Traditional Chinese Medicine, Tianjin, China; ^2^ Tianjin Key Laboratory of TCM Chemistry and Analysis, Institute of Traditional Chinese Medicine, Tianjin University of Traditional Chinese Medicine, Tianjin, China; ^3^ Faculty of Pharmaceutical Sciences, Toho University, Funabashi, Japan

**Keywords:** paeonol, metabolite, pharmacokinetics, tissue distribution, excretion, UPLC-MS/MS

## Abstract

Paeonol is a major bioactive ingredient in Moutan Cortex (the root barks of *Paeonia suffruticosa* Andrews) and exhibited a wide range of bioactivities such as anti-inflammation, anti-oxidation, hypoglycemic effect, analgesic, and others. Even though paeonol has been proven to possess significant pharmacological and therapeutic effects, its pharmacokinetic properties are not satisfactory since it has been found to have a rapid clearance *in vivo*. In the present study, the pharmacokinetics, tissue distribution and excretion of paeonol and its major metabolites were investigated in rats by an efficient and specific UPLC-MS/MS method. The results indicated that paeonol was rapidly absorbed, extensively metabolized, and widely distributed in various tissues without long-term accumulation after oral administration to rats. The major distribution tissues of paeonol and its metabolites were kidney, liver, and heart. Paeonol was able to cross the blood-brain barrier but rapidly decreased after 10 min. The total excretion of four metabolites in urine, bile, and feces was approximately 35.0% within 24 h, and the metabolites were mainly excreted through the urine. In addition, the hypoglycemic activities of paeonol and its metabolites were investigated by a glucose uptake assay on TNF-α mediated insulin resistance in 3T3-L1 adipocytes. The results showed that paeonol and its major metabolites displayed hypoglycemic activities. This is the first comprehensive and systematic report on the pharmacokinetics of paeonol and its metabolites. This research provides an important basis for the clinical development and application of active metabolites.

## Introduction

Paeonol (2'-hydroxy-4'-methoxyacetophenone) is a representative bioactive ingredient in Moutan Cortex (the root barks of *Paeonia suffruticosa* Andrews), one of the most well-known traditional Chinese medicines (Wu et al., 2010). Pharmacologically, paeonol possesses a wide range of bioactivities, highlighted as anti-oxidative (Hsieh et al., 2006), anti-inflammatory (Liu et al., 2017; Lou et al., 2017), anti-thrombotic (Ye et al., 2016), anti-hypertensive (Zhang et al., 2013), anti-diabetic effects (Lau et al., 2007), and anti-neoplastic activities (Xu et al., 2017). Recently, investigations into the effects of paeonol as an anti-diabetic are gaining attention, paeonol was confirmed as the active constituent for inhibiting BBMV glucose uptake and improved oral glucose tolerance *in vivo* (Lau et al., 2007). In addition, studies have shown that paeonol could improve the pathological damage of diabetic encephalopathy in STZ-induced diabetic rats, it might be associated with the modulating AGEs/RAGE/NF-κB pathway (Liu et al., 2013).

In previous studies, the pharmacokinetic of paeonol have been reported after intravenous (2.5–59.2 mg/kg) (Tsai et al., 1994) and oral administration (14.8–140 mg/kg) (Xie et al., 2008), as well as after oral administration of herbal products or TCM prescription containing paeonol (equivalent to 17.5–100 mg/kg) (Xiao et al., 2008; Xie et al., 2014), which have revealed that paeonol has a short half-life *in vivo* and an oral bioavailability of about 16% (Kwon et al., 2017). Additionally, studies on the distribution of paeonol suggested that it was rapidly distributed and eliminated after oral administration, as well as mainly distributed in the liver and kidneys (Li et al., 2011). However, the pharmacokinetic properties of paeonol are not satisfactory to explain the significant pharmacological and therapeutic effects, since paeonol has been found to have a rapid clearance *in vivo*. In contrast, an extensive presence of paeonol metabolites has been identified in rats during our previous investigations (Ding et al., 2017). Moreover, some of the metabolites have exhibited paeonol-related bioactivities, such as anti-inflammatory and anti-oxidant effects (Jin et al., 2016). These facts indicated that a pharmacokinetic investigation of paeonol on the metabolites level is critical for understanding the pharmacological and clinical effectiveness of paeonol.

**Figure 1:**
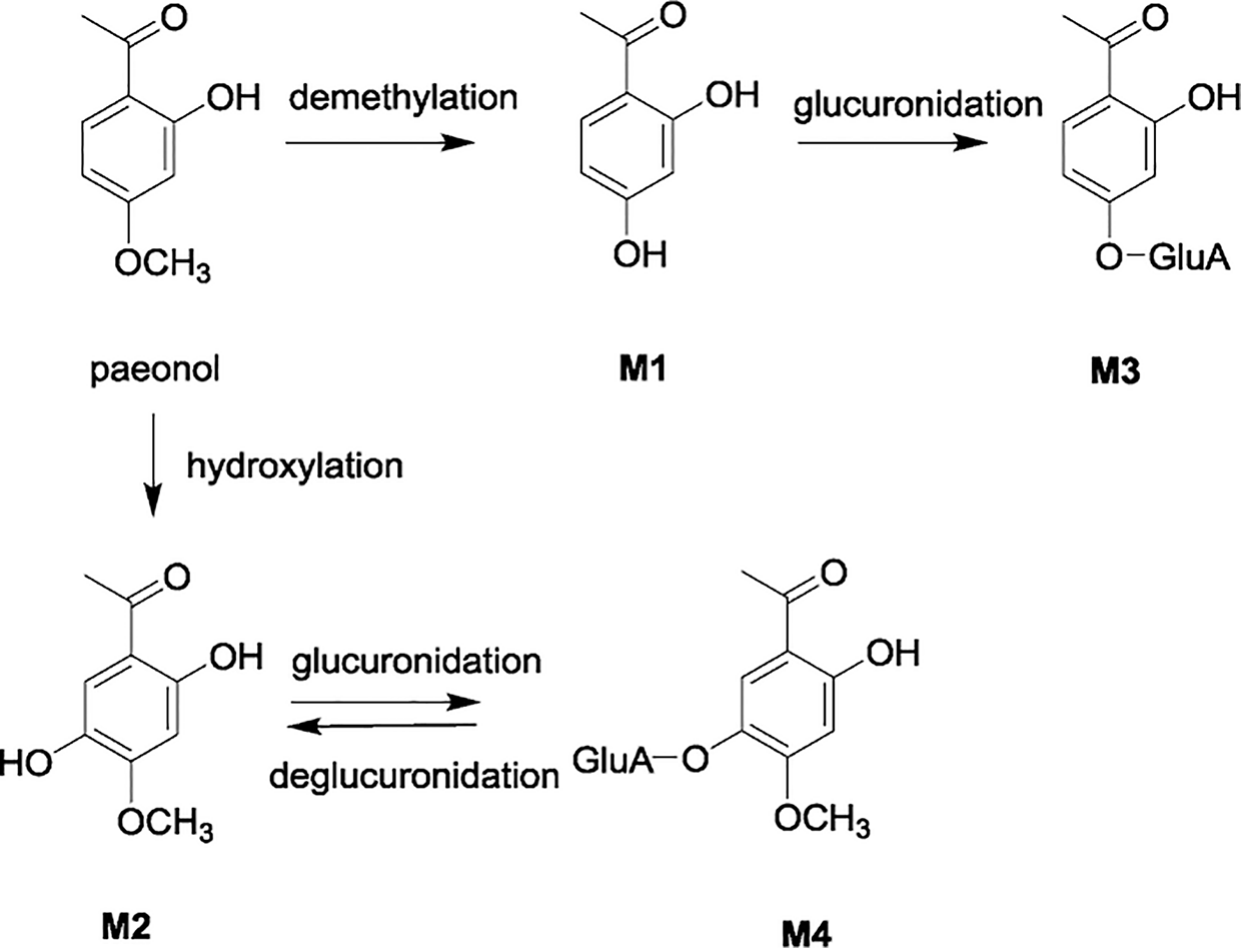
Metabolic pathway of paeonol and its major metabolites M1 – M4.

In the present study, a systematic and comprehensive investigation of the pharmacokinetics, excretion, and tissue distribution of paeonol and its four major metabolites selected, namely, 2,4-dihydroxyacetophenone (**M1**), 2,5-dihydroxy-4-methoxyacetophenone (**M2**), 2-hydroxyacetophenone-4-*O*-glucuronide (**M3**), and 2-hydroxy-4-methoxyacetophenone-5-*O*-glucuronide (**M4**) were carried out after oral administration of paeonol to rats (**Figure 1**). Moreover, the glucose-lowering activities of paeonol and its metabolites were investigated in insulin resistant 3T3-L1 adipocytes.

## Materials and Methods

### Reagents and Chemicals

Paeonol (lot: 20170105, purity > 98%) was purchased from Heng-Yuan Technology Co., Ltd. Tianjin Vientiane (Tianjin, China). Caffeic acid (lot: Y09J8C28349, purity > 98%), the internal standard (IS), was purchased from Yuanye Biotechnology Co., Ltd. (Shanghai, China). 2,4-Dihydroxyacetophenone (**M1**) (lot: C10012347, purity > 98%) was purchased from Shanghai Macklin Biochemical Co., Ltd. (Shanghai, China). 2,5-Dihydroxy-4-methoxyacetophenone (**M2**) (lot: DST181031-059, purity > 99%) was purchased from Chengdu Esite Biotechnology Co., Ltd. (Chengdu, China). 2-Hydroxyacetophenone-4-*O*-glucuronide (**M3**) and 2-hydroxy-4-methoxyacetophenone-5-*O*-glucuronide (**M4**) (purities were > 98% by ^1^H-NMR spectroscopic data, HPLC-PDA, and LC-MS analyses) were prepared in our laboratory (Ding et al., 2012). Acetonitrile and methanol were HPLC grade from Fisher Scientific (Fair Lawn, USA), while ultrapure water was provided by Watsons Group (Hebei, China).

Mouse preadipocytes (3T3-L1) were purchased from the Institute of Basic Medicine of Beijing Academy of Medical Sciences. 3-isobutyl-1-methylxanthine (IBMX), dexamethasone, pioglitazone, TNF-α, EDTA, and 3-(4,5-dimethylthiazol-2-yl)-2,5-diphenyltetrazolium bromide (MTT) were purchased from Sigma (St. Louis, MO, USA). High Glucose (DMEM), fetal calf serum, fetal bovine serum were obtained from Life Technologies (New York, NY, USA). Streptomycin was purchased from Shandong Lukang Pharmaceutical Co., Ltd. (Shandong, China). Glucose oxidase kit was purchased from Beijing Pulilai Gene Technology Co., Ltd. (Beijing, China).

### Animals, Drug Administration, and Sampling

Male Sprague Dawley rats (weighted 200–220 g, aged 5–7 weeks) were purchased from Beijing Vital River Laboratory Animal Technology Co., Ltd. (certificate number: 11400700289812). All the studies on animals were in accordance with the Guidelines for the Care and Use of Laboratory Animals. The animal protocol was approved by the Institutional Animal Care and Use Committee (IACUC) at Tianjin International Joint Academy of Biomedicine.

For pharmacokinetic study, rats were divided into three groups (*n* = 6). Paeonol was suspended in 0.5% CMC-Na aqueous solution, and was given orally to three groups in doses of 12.5 mg/kg, 25 mg/kg, and 50 mg/kg, respectively. For each group, blood samples were collected from the eye venous plexus prior to dosing and at 0.0833, 0.167, 0.25, 0.50, 0.75, 1, 1.5, 2, 4, 6, 8, 12, and 24 h after oral dosing. At each time point, the blood sample (around 0.3 ml) was collected in a tube containing EDTA-2Na (80 mg/ml in physiological saline solution, 10 μl). After centrifuging at 5868 × g for 10 min, the plasma was stored at -80°C until analysis.

For excretion study, rats were randomly and equally divided into two groups (*n *= 18 for each group): one group for evaluating urine and feces, and the other group for evaluating bile excretion. Each group of rats was further divided into three sub-groups (*n* = 6 for each sub-group), and administered intragastrically with different doses of paeonol. For the urinary and fecal excretion, the rats kept in cages were administrated intragastrically with paeonol at doses of 12.5 mg/kg, 25 mg/kg, and 50 mg/kg suspended in 0.5% CMC-Na aqueous solutions, respectively. The metabolic cages allowed the separate collection of urine and feces. Urine samples were collected at 0–4, 4–8, 8–12, 12–24, 24–36, and 36–48 h post-dose and fecal samples were collected at 0–12, 12–24, 24–36, and 36–48 h post-dose. All rats had free access to food and water during the experiments. For biliary excretion, the rats were treated as previously described. After biliary cannulation, the bile samples were collected at 0–2, 2–4, 4–8, 8–12, and 12–24 h post-dose. The bio-samples were stored at –80°C until analysis after recording the excretion volume of urine and bile, and dry weight of the feces for each collection period.

For tissue distribution study, rats were randomly divided into eight groups (*n* = 6). Seven groups were given paeonol (25 mg/kg) orally, and the blank group was given the same volume of 0.5% CMC-Na aqueous solution orally. After the administration, heart, liver, spleen, lung, kidney, and brain samples were collected at 0.167, 0.5, 1, 1.5, 2, 3, 6 h, respectively. Tissue samples were rinsed with 0.9% physiological saline solution to remove the blood and blotted dry with filter paper, accurately weighed, and stored at –80°C until analysis.

### Sample Processing

Plasma protein precipitation method was used for the sample pretreatment. 50 μl of plasma samples were spiked with 200 μl of acetonitrile containing 10 ng/ml of IS. The mixture was vortexed for 2 min and centrifuged at 17,460 × g for 10 min. An aliquot (100 μl) of the supernatant was transferred into another 1.5 ml centrifuge tube and then diluted with 100 μl of distilled water. The each sample was vortexed for 2 min, and 2 μl of supernatant was injected into the UPLC-MS/MS system for analysis.

Each weighed tissue sample was thawed and homogenized in ice-cold physiological saline (1:3, w/v). Then a 200 μl of tissue homogenate was centrifuged at 5868 × g for 10 min, and a 50 μl of supernatant was taken and processed further with the same procedure as those of the plasma samples. Fecal samples were smashed and mixed well, ultrasonically extracted with ten volumes (w/v) of methanol for 30 min to afford fecal sample extracts. Urine, feces, and bile samples were extracted with acetonitrile to precipitate proteins, respectively. The specific treatment was consistent with those of plasma samples. It's worth noting that urine and bile samples were analyzed for each collection interval, while fecal sample was pooled across two separate 0–24 and 24–48 h collection intervals prior to analysis.

### UPLC-MS/MS Condition

An AB Sciex Triple Quad 5500 mass spectrometer (AB Sciex, USA) was used for quantitative analysis. Multiple reaction monitoring (MRM) scans with negative mode was used for detecting the concentration of paeonol and its metabolites in plasma, bile, urine, feces, and various tissues in rats. Mass spectrometer parameters were set as follows: Curtain gas: 30.00 psi; collision gas: 10.00 psi; spray voltage: –4500.00 V; temperature: 500.00°C; atomization gas: 50.00 psi; ion source gas2: 50.00 psi. Optimized multiple reaction monitoring (MRM) parameters for the detection of analytes and internal standard were shown in **Table 1**.

**Table 1:** Optimized multiple reaction monitoring (MRM) parameters for the detection of analytes and internal standard.

Analytes	Q1/Q3	DP	EP	CE	CXP
paeonol	164.9/122.0	–90	-10	-30	-15
**M1**	150.9/135.2	–90	-10	-24	-15
**M2**	181.0/122.9	–90	-10	–40	–15
**M3**	327.2/151.1	–90	–10	–35	–15
**M4**	357.1/165.9	–90	–10	–40	–15
IS	178.9/134.0	–90	–10	–30	–15

Ultra Performance Liquid Chromatography (Waters ACQUITY UPLC, USA) System consisted of the binary pump system, auto-sampler, and column oven. The separation was accomplished by using a C18 column (2.1 × 50 mm, 1.7 μm, ACQUITY UPLC BEH C_18_, Waters, USA). The mobile phase was composed of water-formic acid (100:0.1, v/v) (solvent A) and acetonitrile (solvent B) with a gradient elution: 0–0.5 min, 5.0% B; 0.5–1.0 min, 50% B; 1.0–1.8 min, 50% B; 1.8–2.2 min, 95% B; 2.2–3.8 min, 95% B; 3.8–4.0 min, 5% B; 4.0–5.0 min, 5% B. The flow rate was 0.3 ml/min. The column oven temperature was set at 30°C while sample chamber temperature was set at 12°C, the injection volume was 2 μl.

### Preparation of Stock Solutions and Working Solutions

**M2**, **M3** and **M4** were respectively dissolved in 50% methanol at 1 mg/ml, while paeonol was at 2 mg/mL, and **M1** was at 0.5 mg/ml. 10 μl aliquot of primary paeonol and its four metabolites were spiked in 950 μl 50% acetonitrile. Using aforementioned mixed stock solutions, the working solutions of paeonol and its metabolites were prepared by stepwise dilution. The internal standard working solution was added to an acetonitrile solution containing 0.1% formic acid, prepared to a concentration of 10 ng/ml. All the standard solutions were stored at –20°C until use.

### Preparation of Calibration Curves and Quality Control Samples

Calibration curves were prepared by adding 10 μl of the corresponding working solution and 200 μl of acetonitrile (containing 0.1% formic acid and 10 ng/ml of IS) to 50 μl blank plasma followed by the extraction process to reach the final concentrations. The concentration ranges were 100–3000 ng/ml for paeonol, 50–1,500 ng/ml for **M2**, **M3**, and **M4**, and 5–150 ng/ml for **M1**. Likewise, quality control (QC) samples were prepared independently and similarly to calibration curves at low, medium, and high concentrations of 300, 1200, 2400 ng/ml for paeonol, 150, 600, 1200 ng/ml for **M2**, **M3**, and **M4**, and 15, 60, 120 ng/ml for **M1**.

Regarding tissues (heart, liver, spleen, lung, kidney, brain), urine, bile, and feces samples, a series of working solution were added to the corresponding blank biological matrix of tissue, urine, bile and feces to prepare relevant standard curves and quality control samples. For tissue distribution: the concentration ranges were 10–200 ng/ml for **M1**, 100–2000 ng/ml for paeonol, **M2**, **M3**, and **M4**. LQC, MQC, and HQC were 20, 80, 160 ng/ml for **M1**, respectively, and 200, 800, 1600 ng/ml for paeonol, **M2**, **M3**, and **M4**. As for excretion: the concentration ranges were 100–3000 ng/ml for paeonol, 50–3000 ng/ml for **M1**, **M2**, **M3**, and **M4**. LQC, MQC, and HQC were 300, 1200, 2400 ng/ml for paeonol, respectively, and 150, 1200, 2400 ng/ml for **M1**, **M2**, **M3**, and **M4**.

### Method Validation

The developed method was validated and its specificity, linearity, sensitivity, accuracy, precision, recovery, matrix effect, and stability were confirmed. The validation of bioanalytical assay was carried out in accordance with the procedure of the US FDA guidelines.

### Selectivity

The selectivity was evaluated by comparing the chromatograms of drug-free blank plasma from six rats with the blank plasma spiked with analytes (paeonol and its four metabolites) and IS to exclude any potential interference of endogenous substances.

### Linearity and Lower Limit of Quantification (LLOQ)

The calibration curve was built by assaying the standard plasma samples and plotting the ratio of analyte/IS peak area versus nominal concentration spiked in plasma. The calibration curves used a 1/*X* ^2^ weighted linear regression form. The correlation coefficient > 0.99 was considered to be acceptable. LLOQ was quantified with acceptable accuracy (80%–120%) and precision (< 20%).

### Precision and Accuracy

The reproducibility of accuracy and precision in intra-day and inter-day were assessed by using QC samples at four concentration levels (LLQC, LQC, MQC, and HQC). Analyzing replicates (*n* = 6) at four concentration levels were evaluated over three consecutive days. The concentration of six replicates were used to calculate accuracy, which was expressed as relative error (RE) and was within ±15% of nominal value with the exception of LLOQ, and its deviation did not exceed 20%. Precision was expressed as relative standard derivation (RSD), which was within ± 15% except at LLOQ within ±20%.

### Matrix Effect and Extraction Recovery

To investigate the matrix effect (ME), the analytes and IS were spiked into the blank plasma which were extracted from untreated rats. The matrix effect was determined by comparing the peak area of the analytes spiked into the extracted plasma with the standard solution at the same concentration (*n* = 6). The extraction recovery was evaluated by contrasting the mean peak areas of regular QC samples (*n* = 4) to the values of post-extraction spiked QC samples (*n* = 4).

### Stability

Stability of analytes was determined by analyzing QC samples (*n* = 3) under the following conditions: processed samples placed under the auto-sampler conditions (10°C) for 24 h (post-preparative stability); analytes in rats plasma exposed at room temperature for 6 h (short-term stability); storage of the plasma sample at –80°C for 30 days (long-term stability); three freeze (–80°C) and thaw (25°C) cycles prior to extraction.

### Pharmacokinetic Data and Statistical Analysis

The pharmacokinetic parameters including elimination half-life (*t*_1/2_), mean residence time (MRT), the maximum plasma concentration (*C*_max_), time to reach maximum plasma concentration (*T*_max_), and the area under plasma concentration-time curve (AUC) were calculated based on non-compartmental analysis by DAS 3.0 pharmacokinetic software (Chinese Pharmacology Society). Results were expressed as the mean ± standard deviation (SD).

### Cell Culture and Glucose Uptake Assay

The glucose uptake study was performed according to method (Adam et al., 2016; Li et al., 2017). Briefly, the 3T3-L1 adipocytes were cultured in DMEM supplemented with 10% FBS and1% antibiotic solution (10,000 U/ml penicillin G, 10mg/ml streptomycin) in 5% CO_2_ at 37 ℃, and changed every 48 h. Up to more than 80% of the cells differentiated and matured. For this study insulin resistance in 3T3-L1 was induced by incubation with TNF-α (10 ng/ml) for 96 h, After that, the medium was removed and the cells were treated with paeonol and its metabolites and pioglitazone (Pio, positive control) at 10 μM for 24 h. The supernatant was detected by a glucose oxidase assay kit (Applygen, China). The cells were washed once with PBS (0.01 mol/L, pH 7.4). Then, 100 μl of 5 mg/ml MTT was added to each well, incubated at 37°C for 2.5 h in a 5% CO_2_ incubator, dissolved in 150 μl of DMSO, and measured at 490 nm for detecting cell viability. Cell viability was calculated according to this formula:

$$\text{Cell viability (}\%)=\left[\left({\text{A}}_{490,\text{ sample}}-{\text{A}}_{490,\text{ Blank}}\right)\right]/\left[\left({\text{A}}_{490,\text{control}}-{\text{A}}_{490,\text{Blank}}\right)\right]\times 100\%$$

### Results

#### Establishment of UPLC-MS/MS Method for Simultaneous Quantitative Analysis of Paeonol and Selected Metabolites

A UPLC-MS/MS method was successfully established for simultaneous quantitative analysis of paeonol and four selected major metabolites **M1** ~ **M4**. Key items of optimization were summarized as follows. The plasma samples treated with acetonitrile (containing 0.1% formic acid) provided stable and reproducible results. Mobile phase of acetonitrile-water yielded satisfactory retention performances for all analytes together with IS, and addition of 0.1% formic acid to water increased ESI efficiency as well as further optimize the chromatographic pattern. Negative ionization mode provided better mass responses than positive mode.

**Figure 2:**
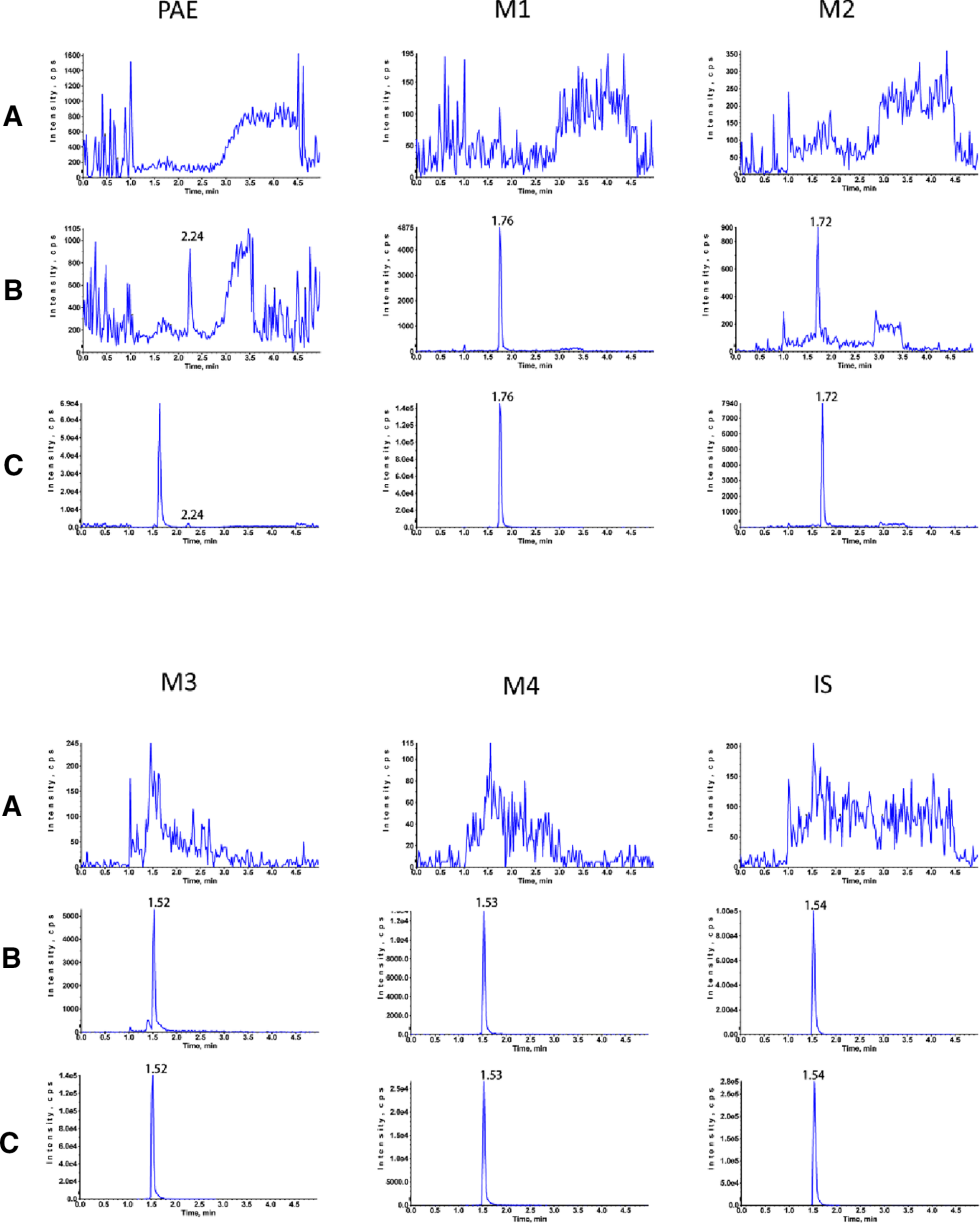
Typical MRM chromatograms of rat plasma. **(A)** blank rat plasma sample; **(B)** Blank rat plasma spiked with standard solutions; **(C)** plasma sample of 5 min after oral administration of 25 mg/kg paeonol.

All the results of bioanalytical assay validation were satisfactory as specified in US FDA guidelines. Typical chromatograms of blank plasma, blank plasma spiked with analytes and IS, and plasma sample after oral administration are illustrated in **Figure 2**. The target analytes were eluted at 2.24 min (paeonol), 1.76 min (**M1**), 1.72 min (**M2**), 1.52 min (**M3**), 1.53 min (**M4**), and 1.54 min (IS). Linear regression curves showed good linearity and the correlation coefficients (*r* ^2^) were over 0.994 in all analytes. In plasma, the RSDs of precision ranged from 1.10% to 8.14% for the intra-day and 2.36% to 10.82% for the inter-day, and the accuracy was within ±14.03% for both the intra- and inter-day. The matrix effect ranged from 83.37% to 115.18%, and the mean recoveries of five analytes ranged from 85.29% to 103.30%.

The results indicated that the recoveries of the analytes were high and constant by using current sample treatment methods, the RSDs of five analytes were within 13.32%. The stability of analytes in rats plasma under different conditions were evaluated at three concentration levels in three replicates, including post-preparative stability, short-term stability, long-term stability, and freeze-thaw cycles stability. The results showed that the RSDs% varied from 0.48% to 8.31%, therefore, all the samples were stable under various conditions.

#### Pharmacokinetic Study

**Figure 3:**
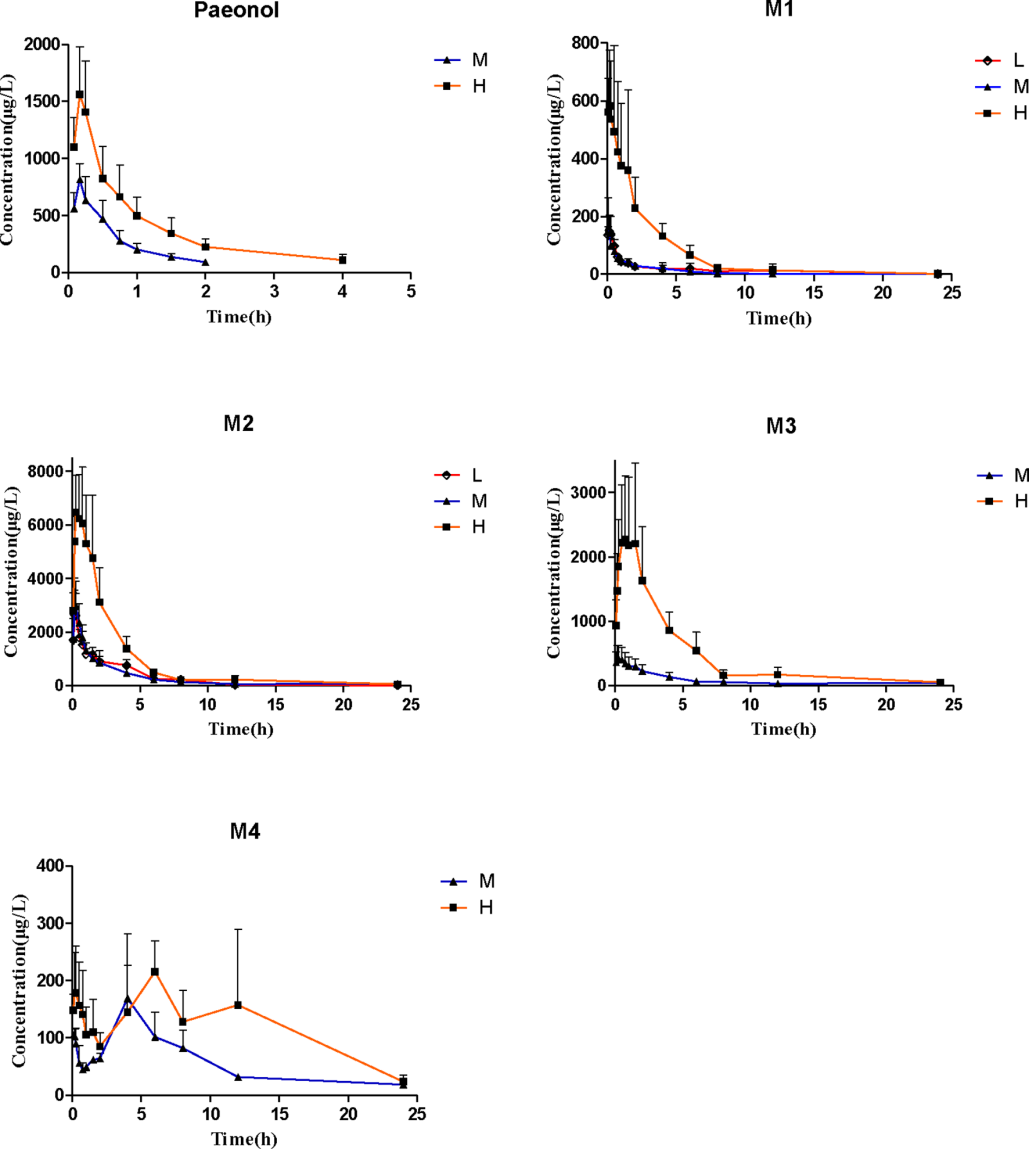
Rat plasma concentration-time course after receiving 12.5 mg/kg, 25 mg/kg, and 25 mg/kg paeonol by oral administration. (Mean ± SD, *n* = 6).

The validated assay method was successfully applied to a pharmacokinetic study of simultaneous determination of paeonol and its four metabolites in rat plasma following oral doses of 12.5 mg/kg, 25 mg/kg, and 50 mg/kg. The mean plasma concentration-time curves and the pharmacokinetic parameters based on non-compartmental model are provided in **Figure 3** and **Table 2**.

**Table 2:** Pharmacokinetic parameters of analytes after oral administration of paeonol to rats.

Analytes	Dose (mg/kg)	*V*/*F* (L)	*t*_1/2_ (h)	*T*_max_ (h)	*C*_max_ (μg/L)	AUC_(0-_ *_t_*_)_ (μg/L*h)	AUC_(0-∞)_ (μg/L*h)	MRT_(0-t)_ (h)
paeonol	12.5	—	—	—	—	—	—	—
	25.0	31.69 ± 9.72	0.68 ± 0.22	0.18 ± 0.03	825.86 ± 150.06	539.78 ± 165.52	644.00 ± 174.00	0.55 ± 0.11
	50.0	21.84 ± 13.23	1.26 ± 1.13	0.19 ± 0.04	1588.71 ± 419.90	1332.76 ± 413.41	1654.22 ± 558.14	0.85 ± 0.38
**M1**	12.5	297.32 ± 156.06	3.33 ± 2.42	0.14 ± 0.10	154.33 ± 49.60	270.72 ± 144.15	325.04 ± 163.96	2.41 ± 1.67
	25.0	285.93 ± 70.25	2.22 ± 0.66	0.12 ± 0.04	155.00 ± 93.85	201.63 ± 86.81	233.41 ± 84.44	1.80 ± 0.43
	50.0	43.11 ± 22.35	2.10 ± 0.97	0.24 ± 0.21	635.67 ± 223.35	1474.47 ± 523.82	1541.54 ± 519.67	2.49 ± 0.41
**M2**	12.5	9.92 ± 2.02	2.21 ± 0.27	0.22 ± 0.05	2830.00 ± 962.34	6271.13 ± 1770.92	6688.89 ± 2060.09	2.72 ± 0.49
	25.0	9.19 ± 6.35	1.94 ± 1.25	0.20 ± 0.04	3155.71 ± 1004.07	5872.50 ± 1340.72	6277.51 ± 1976.69	3.19 ± 1.40
	50.0	4.39 ± 3.04	2.90 ± 2.40	0.50 ± 0.29	6844.29 ± 1630.09	18292.59 ± 5854.16	18568.62 ± 5870.21	2.60 ± 0.50
**M3**	12.5	—	—	—	—	—	—	—
	25.0	23.78 ± 7.28	1.59 ± 0.90	0.56 ± 0.72	466.00 ± 213.53	1706.39 ± 477.94	1860.22 ± 573.75	6.51 ± 3.05
	50.0	7.92 ± 5.19	2.44 ± 1.24	0.79 ± 0.37	2497.83 ± 1142.88	9819.12 ± 3955.40	10003.57 ± 3981.12	3.80 ± 1.26
**M4**	12.5	—	—	—	—	—	—	—
	25.0	108.64 ± 77.88	3.67 ± 1.84	5.50 ± 1.00	153.25 ± 62.65	1388.43 ± 499.10	1374.49 ± 486.22	8.28 ± 2.45
	50.0	59.52 ± 27.03	4.77 ± 2.10	4.06 ± 2.71	242.25 ± 86.70	1655.53 ± 811.52	2434.51 ± 613.28	5.94 ± 0.60

“—” means undetected.

(Mean ± SD, n=6).

#### Tissue Distribution Study

**Figure 4:**
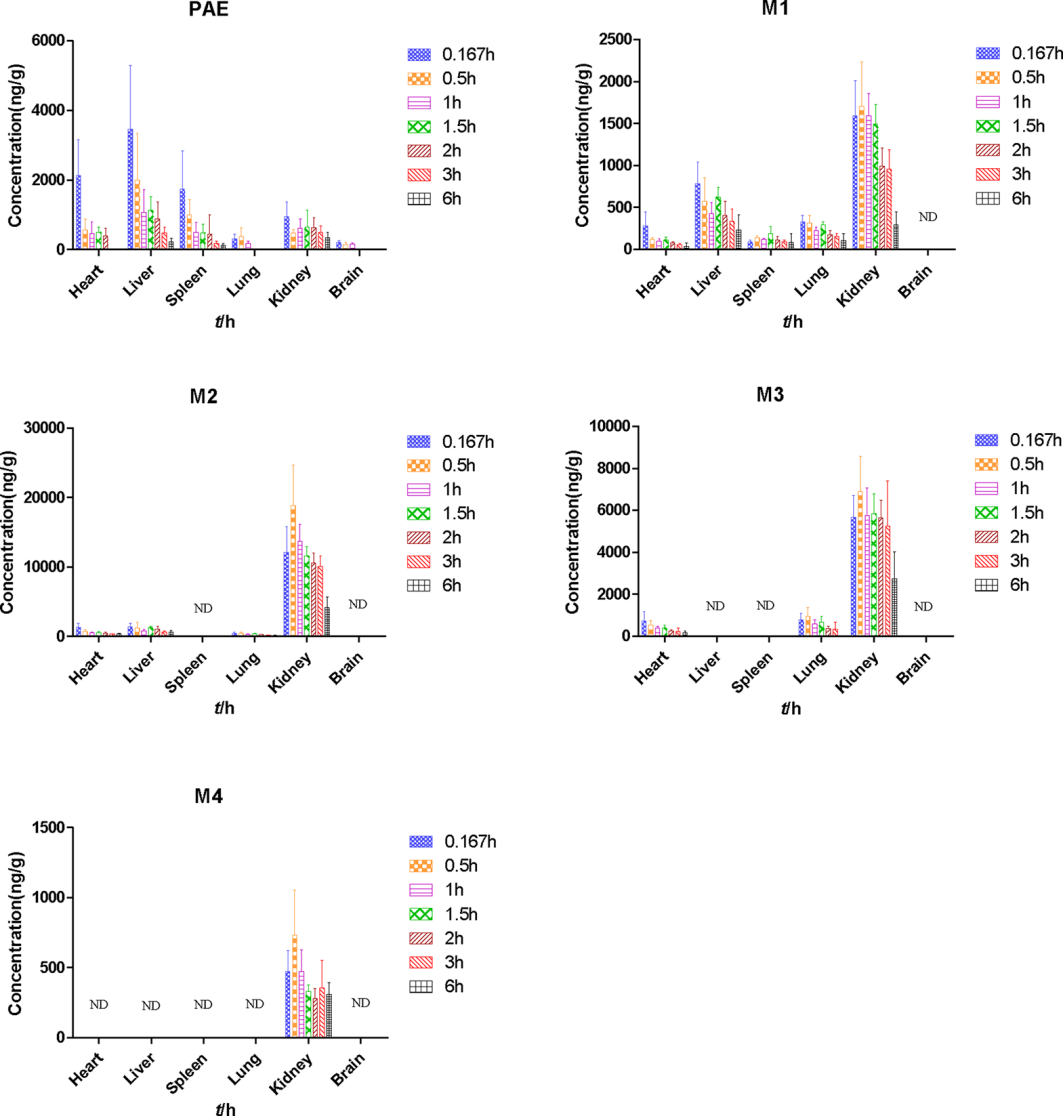
Tissue distribution at different time points after oral administration of 25 mg/kg paeonol in rats. (Mean ± SD, *n* = 6).

After the administration of 25 mg/kg paeonol, heart, liver, spleen, lung, kidney, and brain samples were collected at 0.167, 0.5, 1, 1.5, 2, 3, 6 h, respectively. Tissue distribution at different time points after oral administration in rats was shown in **Figure 4** and **Table 3**.

**Table 3:** Pharmacokinetic parameters of analytes in different tissues after oral administration of 25 mg/kg paeonol to rats.

Analytes	Tissue	*t*_1/2_ (h)	*T*_max_ (h)	*C*_max_ (μg/L)	AUC_(0-_ *_t_*_)_ (μg/L*h)	AUC_(0-∞)_ (μg/L*h)
paeonol	Heart	—	—	—	—	—
	Liver	2.27 ± 0.83	0.28 ± 0.16	887.29 ± 240.42	1854.19 ± 504.32	2282.02 ± 245.85
	Spleen	—	—	—	—	—
	Lung	—	—	—	—	—
	Kidney	2.09 ± 0.96	0.93 ± 0.87	1113.29 ± 343.63	2691.36 ± 420.94	3620.02 ± 909.29
	Brain	—	—	—	—	—
**M1**	Heart	2.61 ± 1.58	0.40 ± 0.50	296.43 ± 146.28	261.81 ± 77.47	649.36 ± 281.50
	Liver	2.27 ± 0.83	0.45 ± 0.49	877.29 ± 240.412	2282.02 ± 245.85	2854.19 ± 504.32
	Spleen	3.36 ± 1.27	1.70 ± 0.24	238.86 ± 65.01	625.56 ± 167.78	1064.02 ± 574.00
	Lung	2.53 ± 1.26	0.28 ± 0.16	383.67 ± 46.57	1048.77 ± 126.40	1304.79 ± 366.17
	Kidney	2.28 ± 0.99	0.76 ± 0.58	1980.00 ± 371.84	6005.228 ± 1044.66	8069.93 ± 3855.56
	Brain	—	—	—	—	—
**M2**	Heart	4.28 ± 1.15	0.40 ± 0.50	1310.86 ± 509.26	2693.23 ± 244.35	4637.81 ± 1402.73
	Liver	2.78 ± 0.71	0.71 ± 0.76	2115.00 ± 472.31	4153.52 ± 987.94	6166.20 ± 1739.99
	Spleen	2.27 ± 1.40	0.33 ± 0.17	581.00 ± 115.30	1080.05 ± 274.57	1464.05 ± 424.29
	Lung	—	—	—	—	—
	Kidney	3.01 ± 0.67	0.33 ± 0.17	18800.00 ± 4109.34	49511.34 ± 10521.47	76840.00 ± 12461.65
	Brain	—	—	—	—	—
**M3**	Heart	2.17 ± 1.12	0.64 ± 0.60	889.57 ± 293.47	1297.69 ± 601.50	1594.42 ± 440.91
	Liver	—	—	—	—	—
	Spleen	—	—	—	—	—
	Lung	1.53 ± 1.36	0.33 ± 0.17	1204.33 ± 242.22	1446.14 ± 197.74	2083.37 ± 673.22
	Kidney	4.29 ± 2.90	0.44 ± 0.12	7483.33 ± 1051.74	25652.59 ± 4750.48	47606.49 ± 28384.46
	Brain	—	—	—	—	—
**M4**	Heart	—	—	—	—	—
	Liver	—	—	—	—	—
	Spleen	—	—	—	—	—
	Lung	—	—	—	—	—
	Kidney	2.90 ± 1.50	0.93 ± 1.04	833.80 ± 276.67	1952.06 ± 464.96	4302.04 ± 2132.66
	Brain	—	—	—	—	—

“—” means undetected

(Mean ± SD, n=6).

#### Excretion Study

**Figure 5:**
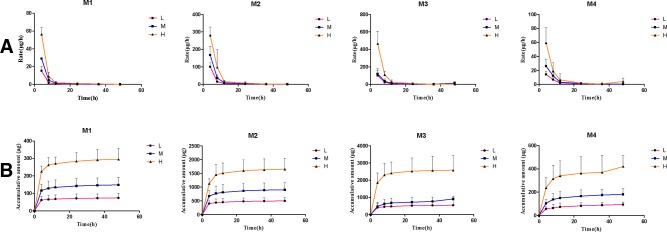
The mean urinary excretion rate-time profile **(A)** and the accumulative amount-time profile **(B)** within 0–48 h of four metabolites. (Mean ± SD, *n* = 6).

**Figure 6:**
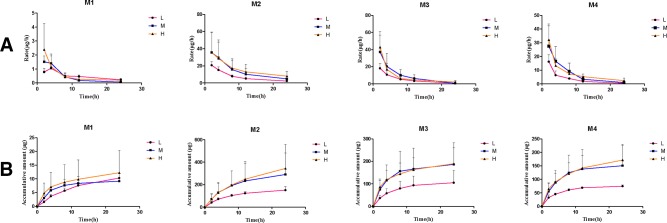
The mean biliary excretion rate-time profile **(A)** and the accumulative amount-time profile **(B)** within 0–48 h of four metabolites. (Mean ± SD, *n* = 6).

After the administration of different doses of paeonol (12.5 mg/kg, 25 mg/kg, and 50 mg/kg) to rats, cumulative excretion in urine, bile, and feces were determined and the results are shown in **Table 4**. The mean urinary and biliary excretion rate-time profile and the accumulative amount-time profile within 0–48 h of four metabolites are shown in **Figures 5** and **6**. In various drug elimination, the concentrations of paeonol were detected below the minimum quantitative limit, this situation may be caused by extensive and rapid metabolism. The total recoveries of four metabolites in urine, bile, and feces were approximately 35.0% within 24 h. The cumulative urinary excretion of metabolites were 29.8%, 26.3%, and 27.4% of different doses for 48 h, the cumulative biliary excretion were 8.24%, 7.64% and 1.76% for 24 h. Meantime, the recoveries of metabolites from rat feces were very small, only 0.03%, 0.04%, and 0.02%.

**Table 4:** The cumulative excretion percentage (mole percent) of all metabolites in urine, bile and feces at different doses.

Biological sample	Dose (mg/kg)	M1 (%)	M2 (%)	M3 (%)	M4 (%)	Total (%)
Urine	12.5	2.81 ± 0.92	15.86 ± 3.66	9.57 ± 4.71	1.51 ± 0.36	29.75
0~48 h	25.0	2.82 ± 0.84	14.31 ± 4.08	8.04 ± 1.47	1.46 ± 0.40	26.63
	50.0	2.56 ± 0.55	11.99 ± 2.92	10.42 ± 3.44	1.55 ± 0.34	26.52
Bile	12.5	0.39 ± 0.01	4.80 ± 0.98	1.85 ± 0.95	1.20 ± 0.07	8.24
0~24 h	25.0	0.17 ± 0.05	4.61 ± 3.03	1.64 ± 0.84	1.22 ± 0.61	7.64
	50.0	0.11 ± 0.07	2.50 ± 1.56	0.77 ± 0.28	0.63 ± 0.21	1.76
Feces	12.5	0.03 ± 0.03	—	—	—	0.03
0~48 h	25.0	0.04 ± 0.02	—	—	—	0.04
	50.0	0.02 ± .0.01	—	—	—	0.02

“—” means undetected.

(Mean ± SD, n=6).

#### Glucose Uptake and MTT Assay of TNF-α—Induced Insulin Resistance 3T3-L1 Adipocytes

**Figure 7:**
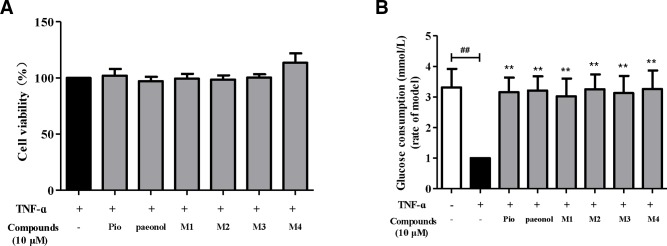
Effects of paeonol and metabolites on the cytotoxicity **(A)** and glucose consumption of insulin resistant 3T3-L1 **(B).** ^##^ *P *< 0.01, compared with the blank control group; **P< 0.01, compared with the model control group.

The results are shown in **Figure 7**, the hypoglycemic activities of paeonol and the metabolites were investigated on TNF-α mediated insulin resistance in 3T3-L1 adipocytes. As a result, paeonol and its metabolites did not show significant toxicity to 3T3-L1 cells at 10 μM. In cellular glucose uptake assay, paeonol and its major metabolites enhanced the insulin-stimulated glucose uptake in insulin-resistance adipocytes. The hypoglycemic activities of paeonol and its metabolites are similar with positive drug (pioglitazone).

## Discussion

In the pharmacokinetics study, after oral administration of different doses (25 mg/kg and 50 mg/kg), paeonol was absorbed quickly into blood with the time to reach *T*_max_ at 0.18 ± 0.03 h and 0.19 ± 0.04 h, and then eliminated from rats plasma with *t*_1/2_ of approximately 0.68 h. Two and four hours after the medium and high dose administration, the concentration of paeonol was lower than LLOQ. These results showed that the total exposure amount (AUC_0-∞_) from large to small in turn was **M2** > **M3** > **M4** > **M1** > paeonol, parent drug was rapidly and extensively metabolized. In addition, **M2** was a phase I metabolite with a high plasma exposure, the AUC_0–∞_ of **M2** calculated as 18568.62 ± 5870.21 μg/l*h in high dose, it was presumable that **M2** may be an active metabolite.

The parent compound and its four metabolites have specific transformation pathways *in vivo*, including demethylation, glucoronidation, hydroxylation, and hydrolysis by gastrointestinal flora (**Figure 1**). It was observed that **M4** presented two maximum concentrations round 5 min and 4.5 h, respectively. This pharmacokinetic profile may be attributed to an enterohepatic circulation, namely, the phase I metabolite **M2** was glucuronidated to produce **M4** in the liver, then secreted into the bile and discharged into the intestine, and further hydrolyzed to **M2** under the action of enterobacteria. Additionally, **M2** was an oxidative metabolite of paeonol, which was absorbed by gastrointestinal tract into the liver again. Similarly, **M3** did not exhibit the double-absorption peak phenomenon in the mean plasma concentration-time curves. There were two possible reasons to explain this phenomenon. Urinary excretion data and plasma exposure of **M3** vs. **M4** seem to indicate that **M3** was primarily excreted *via* renal route compared to **M4**. In addition, the higher exposure of **M3** in plasma may mask the relatively small increase in **M3** *via* enterohepatic recirculation. The non-linear increase in metabolite exposure with increasing dose may be explained by saturation of clearance pathways as indicated by the decrease in biliary clearance and the slight decrease in renal clearance of metabolites with increasing dose.

In the tissue distribution study, the highest concentration level of paeonol was observed in the liver (3460.00 ± 1830.82 ng/g) at 10 min, followed by heart (2130.14 ± 1032.74 ng/g), spleen (1743.83 ± 1090.95 ng/g), kidney (953.14 ± 423.09 ng/g), lung (313.86 ± 128.95 ng/g), and brain (202.86 ± 59.57 ng/g), suggesting that paeonol distributed rapidly and widely in various tissues. At 3 h, the concentration of paeonol in tissue was significantly reduced, which was consistent with plasma pharmacokinetics, indicating that paeonol could be rapidly eliminated and metabolized *in vivo*. The kinetics of **M4** in the tissue level followed plasma concentration, at the time point corresponding to the plasma concentration, there was also a double peak in the concentration of **M4** in the kidney. The phase I metabolites **M1** and **M2** were more widely distributed in tissues than the phase II metabolites **M3** and **M4**. Moreover, the distributed maximum concentrations of phase II metabolites in kidney were higher than others, indicating that the phase II metabolites generated in liver were mainly excreted by the kidney.

It's worth mentioning that the low level of paeonol detected in brain indicated that paeonol could pass the blood-brain barrier, but it was rapidly decreased after 10 min and not detected after 1.0 h. The maximum concentration of paeonol in the brain could reach 202.86 ± 59.57 ng/g. It has been reported paeonol exhibited antidepressant (Zhu et al., 2015; Zhu et al., 2018) and neuroprotective effects (Liu et al., 2019), which requires further study. The average maximum exposures of paeonol, **M1**, **M2,** and **M3** in the heart were 2131.14 ± 1032.74 ng/g, 281.85 ± 163.75 ng/g, 1257.49 ± 578.90 ng/g, and 722.86 ± 444.93 ng/g, respectively. The relative exposures of paeonol and its metabolites in heart with their similar elimination half-life, may provide further support to the reports that paeonol has good curative effect on cardiovascular disease (Li et al., 2015; Wu et al., 2018). In addition, the extensive tissue distribution of paeonol in liver [AUC_(0-∞)_ 2282.02 ± 245.851 (μg/L*h), t_1/2_ 2.27 ± 0.83 h] may add further support to the use of paeonol as a protective strategy against alcoholic liver injury by attenuating hepatic steatosis, inflammatory response, and apoptosis (Hu et al., 2010).

In excretion study, the order of biliary cumulative excretion amount of four metabolites for 24 h was **M2** > **M3** > **M4** > **M1**. From the results, cumulative excretion amount in bile was saturated with the increase in dose. Fecal excretion results showed that only a small amount of **M1** was detected, while the others were undetectable. It is predicted that paeonol was mainly metabolized in the liver, not by intestinal bacteria.

Combined with the high concentration of metabolites in plasma and tissues for this study, it is speculated that the clinical and pharmacological effects of paeonol are produced by the combination of paeonol and its metabolites. Although paeonol is rapidly cleared *in vivo*, it is speculated that there is enterohepatic circulation, which maintain the concentration of metabolites *in vivo* and also significantly prolong the drug action time. However, paeonol was able to cross the blood-brain barrier, which exerted antidepressant and neuroprotective efficacy in brain. In the glucose uptake assay, based upon the observed exposures in plasma and liver and kidney, a 10 µM concentration of paeonol and its metabolite was considered the most appropriate for testing as it was within the observed maximum concentration range. Metabolites **M1**–**M4**, all exhibited similar potency to paeonol, which revealed that not only paeonol but also its metabolites played hypoglycemic activity role by enhancing insulin resistance glucose uptake in 3T3-L1 adipocytes.

## Conclusion

In summary, this is the first and comprehensive report to evaluate the pharmacokinetics, tissue distribution and excretion of paeonol and its metabolites in rats. An accurate, reliable and sensitive UPLC-MS/MS method was validated for simultaneous quantitative analysis of paeonol and its metabolites in rat biological samples such as plasma, urine, bile, feces, and tissue samples. Paeonol is rapidly absorbed, extensively metabolized, and widely distributed in various tissues with no long-term accumulation after oral administration. Pharmacokinetic parameters such as *C*_max_, *T*_max_, and AUC_0-∞_, showed paeonol was rapidly absorbed and eliminated in rats in a dose-dependent manner. The major distribution tissues of paeonol and its metabolites in rats were in liver and kidney. Heart and lung had similar distribution characteristics and contained small amounts of paeonol, **M1**-**M3**. Only paeonol and a few of **M1** could be detected in the spleen. It's worth mentioning that a small amount of paeonol was able to cross the blood-brain barrier. The total excretion of four metabolites in urine, bile and feces was approximately 35.0% within 24 h, metabolites were mainly excreted through the urine. In addition, paeonol and its major metabolites improved TNF-α mediated insulin resistance in 3T3-L1 adipocytes. Therefore, this research provides scientific and reliable support for fully understanding the role of paeonol and its metabolites on hypoglycemic activity which deserve further study.

## Data Availability Statement

All datasets generated for this study are included in the article/supplementary material.

## Ethics Statement

The animal study was reviewed and approved by the Institutional Animal Care and Use Committee (IACUC) at Tianjin International Joint Academy of Biomedicine.

## Author Contributions

XH and LD carried out experimental work, analyzed the data, and wrote the manuscript. SC, LC, KW, and CG participated in the investigation. LD, WL, and KK contributed to the final preparation of this paper and submission. WL and FQ designed and supervised this research.

## Conflict of Interest

The authors declare that the research was conducted in the absence of any commercial or financial relationships that could be construed as a potential conflict of interest.
